# Diagnostic Accuracy of Wireless Capsule Endoscopy in Polyp Recognition Using Deep Learning: A Meta-Analysis

**DOI:** 10.1155/2022/9338139

**Published:** 2022-03-19

**Authors:** Junjie Mi, Xiaofang Han, Rong Wang, Ruijun Ma, Danyu Zhao

**Affiliations:** ^1^Digestive Endoscopy Center, Shanxi Provincial People's Hospital, Taiyuan, China; ^2^Reproductive Medicine, Shanxi Provincial People's Hospital, Taiyuan, China

## Abstract

**Aim:**

As the completed studies have small sample sizes and different algorithms, a meta-analysis was conducted to assess the accuracy of WCE in identifying polyps using deep learning.

**Method:**

Two independent reviewers searched PubMed, Embase, the Web of Science, and the Cochrane Library for potentially eligible studies published up to December 8, 2021, which were analysed on a per-image basis. STATA RevMan and Meta-DiSc were used to conduct this meta-analysis. A random effects model was used, and a subgroup and regression analysis was performed to explore sources of heterogeneity.

**Results:**

Eight studies published between 2017 and 2021 included 819 patients, and 18,414 frames were eventually included in the meta-analysis. The summary estimates for the WCE in identifying polyps by deep learning were sensitivity 0.97 (95% confidence interval (CI), 0.95–0.98); specificity 0.97 (95% CI, 0.94–0.98); positive likelihood ratio 27.19 (95% CI, 15.32–50.42); negative likelihood ratio 0.03 (95% CI 0.02–0.05); diagnostic odds ratio 873.69 (95% CI, 387.34–1970.74); and the area under the sROC curve 0.99.

**Conclusion:**

WCE uses deep learning to identify polyps with high accuracy, but multicentre prospective randomized controlled studies are needed in the future.

## 1. Introduction

Colorectal cancer (CRC) is a common malignant tumour that seriously affects human health, with the 3rd highest incidence and 2nd highest mortality rate of malignant tumours in the world [1, 2]. Almost all CRC originate from colorectal polyps, and regular screening and early detection of polyps are by far the most effective way to prevent CRC [3, 4]. In clinical practice, colonoscopy is a commonly used method to screen for colorectal polyps, but colonoscopy may cause pain and some complications such as bleeding and perforation. In addition, anesthetics are sometimes used in colonoscopy [5, 6]. Wireless capsule endoscopy (WCE) is a new noninvasive endoscopic technique that can overcome some of the shortcomings of colonoscopy. In addition, WCE has a high accuracy rate in screening and diagnosing polyps [7].

A complete WCE can produce over 50,000 images, which are tedious and time consuming for a gastroenterologist to read, taking approximately 50 minutes [8]. Artificial intelligence may be the way to solve the problem of gastroenterologists reading the large number of images generated by WCE. Hand-engineered methods and deep learning are included in artificial intelligence. Among them, hand-engineered methods are a way to identify low-level image-descriptive features of polyps by marking the texture, shape, and color information of the polyps and training the classifier [9–12]. Deep learning can analyse and process data such as images and sounds intelligently by learning the patterns and expressions inherent in a large number of samples, enabling the recognition of data such as images and sounds [13]. With the development of computer technology, deep learning has made significant progress in the field of vision with its unique feature learning capabilities [14]. Currently, most of the WCE studies that use deep learning to identify polyps are proprietary databases, and this lack of data sharing is not conducive to multicentre studies with large samples. In addition, studies are now retrospective and may suffer from selective bias. Many studies have been done on WCE by using deep learning to identify polyps [15–22]. However, the findings of WCE using deep learning to identify polyps are not sufficient due to single-center studies, small sample studies, and different research centers using different methods and algorithms. In this study, we performed a meta-analysis in order to assess the accuracy of WCE in identifying polyps using deep learning.

## 2. Method

### 2.1. Search Strategy

Two authors independently conducted a comprehensive and systematic search of PubMed, Embase, Web of Science, and Cochrane databases, respectively, up to December 8, 2021 (in Supplementary Information Part IV). Search terms include the following: (“convolutional neural network” OR “artificial intelligence” OR “AI” OR “neural networks” OR “computer-aided diagnosis” OR “deep learning”) AND (“colon capsule endoscopic images” OR “colon capsule endoscopy” OR “capsule endoscopy”) AND (“colorectal neoplasia” OR “colon cancer” OR “colonic polyps” OR “colorectal polyps” OR “colorectal polyp screening” OR “colorectal neoplasms”). References to studies retrieved from the database were hand searched as additional sources. The literature search was done independently by two reviewers (Mi and Han), and if differences were encountered, they were discussed by adding a third person (Wang).

### 2.2. Inclusion and Exclusion Criteria

Inclusion criteria: (i) WCE uses deep learning to identify polyps; (ii) the number of true positives (TP), false positives (FP), true negatives (TN), and false negatives (FN) can be obtained directly or indirectly from the study; (iii) the studies included are full text and are not restricted by language; (iv) protruding lesions of the colon were also included in the study, as most protruding lesions are polyps. Exclusion criteria: (i) WCE uses hand-engineered methods to identify polyps; (ii) conference abstracts, letters to editors, reviews, case reports, comments, and editorials.

### 2.3. Data Extraction and Quality Assessment

Data extraction was done independently by two reviewers (Mi and Wang). If there is a disagreement, a third person (Ma) will be added for discussion. The data extracted from the included studies included the following: first author, year of publication, country, no. of patients, dataset size, total dataset size, annotator's experience, method, algorithm, study design, center, speed of frames reading, types of capsule endoscopes, types of databases, test images, and journal type. Qualitative assessment and evaluation of potential bias were performed according to the quality assessment of diagnostic accuracy studies-2 (QUADAS-2) [23].

### 2.4. Statistical Analysis

Assessment of the accuracy of pooled studies to identify polyps includes sensitivity, specificity, positive likelihood ratio (PLR), and negative likelihood ratio (NLR). The area under the sROC curve (AUC) and the diagnostic odds ratio (DOR) are comprehensive indicators to evaluate the accuracy of diagnosis. The clinical applicability of WCE to identify polyps was evaluated using Fagan's plot and the likelihood matrix. The Cochrane Q test, expressed as I^2^, was used to assess the heterogeneity of the included studies, and I^2^ > 50% or *P* < 0.1 was considered significant heterogeneity, prompting the use of the random effects model (DerSimonian–Laird method), otherwise a fixed effects model (Mantel–Haenszel method). To explore the accuracy of WCE using deep learning to identify polyps in different subgroups and possible sources of heterogeneity in the study, a subgroup analysis and meta-regression were performed according to the following: dataset size, no. of patients, total dataset size, country, method, types of capsule endoscopes, test images, and journal type. The present meta-analysis used Deek's test and funnel plot analysis for publication bias. The closer the angle in the Deek's funnel plot between the regression line and the vertical axis is to 90°, the less likely the publication bias is. There is publication bias when *P* < 0.05. To assess the robustness of the synthesized results, sensitivity analyses will be conducted. The Spearman's correlation coefficient was used to assess the threshold effect using Meta-DiSc software version 1.4 (Cochrane Colloquium, Barcelona, Spain). All analyses for the study were performed using STATA software version 16.0 (Stata Corp, College Station, Texas, USA). The quality of the included studies was assessed using Review Manager 5.3 software (Cochrane Collaboration, Oxford, UK). The significance level was measured at *P* < 0.05.

## 3. Results

### 3.1. Included Studies and Quality Assessment

**Figure 1:**
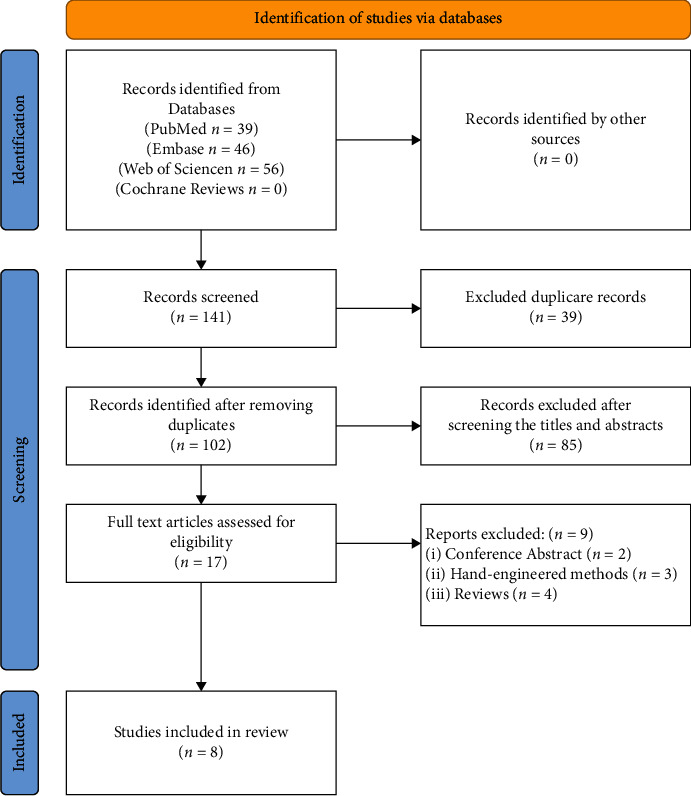
Flow diagram of the published articles evaluated for inclusion in this meta-analysis.

Searching using the preset search strategy resulted in 141 records in Figure 1. After eliminating duplicate records, there were 102 records left. 85 records were excluded immediately after a review of titles and abstracts. After reading the full text of the remaining records, 9 records were further excluded for various reasons. Eight studies published between 2017 and 2021 were finally included in the meta-analysis in Figure 1. In total, there were 819 patients and 18,414 frames in 8 studies in Table 1. The quality of the included studies was assessed using the QUADAS-2 assessment tool (in Supplementary Information Part III).

### 3.2. Diagnostic Performance and Clinical Applicability

**Figure 2:**
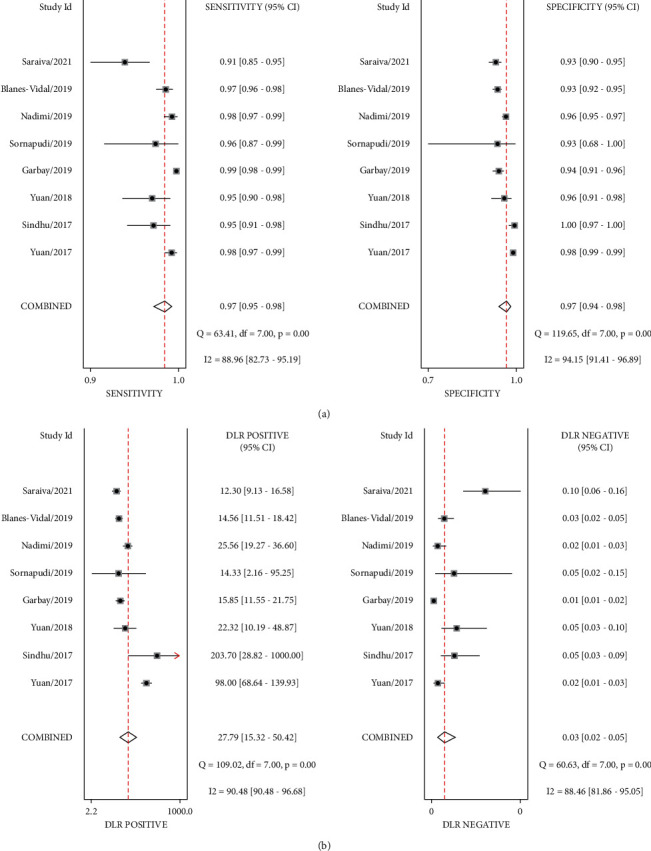
Forest plot of sensitivity, specificity, PLR, and NLR of WCE in identifying polyps; (a) sensitivity and specificity and (b) PLR and NLR.

**Figure 3:**
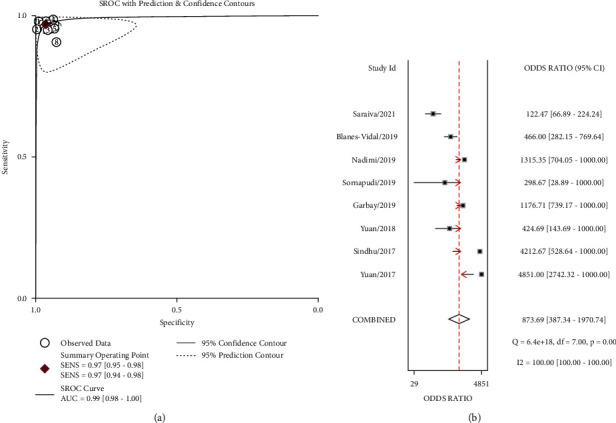
SROC plot and DOR on WCE using deep learning to identify polyps; (a) SROC plot on WCE using deep learning to identify polyps. Each circle indicates an individual study; red diamond represents summary sensitivity and specificity; inner and outer ellipses indicate 95% confidence region and prediction region, respectively. (b) The DOR suggests how much higher the odds of having the polyps are for the people with a positive test result than those with a negative test result. The diamond represents the pooled DOR.

**Figure 4:**
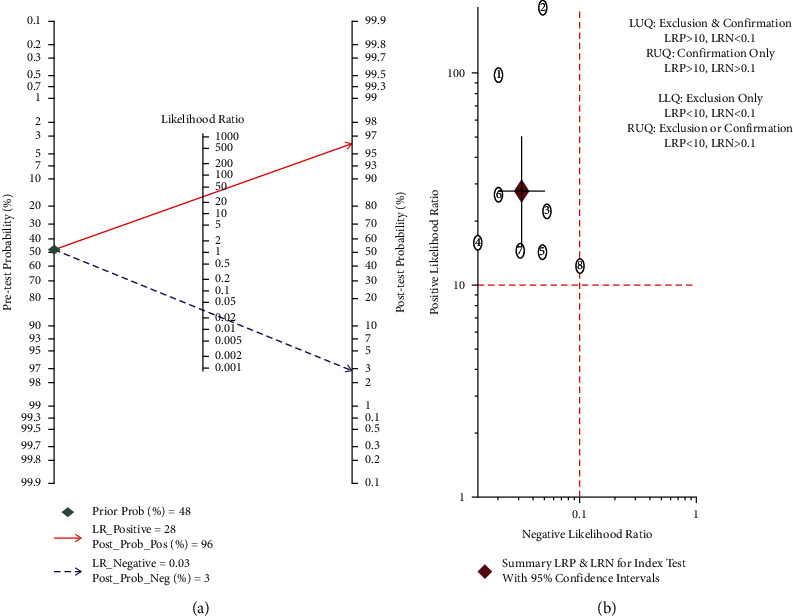
(a) The Fagan plot depicts the impact of WCE using deep learning on pretest probabilities (the pooled prevalence of polyps, along with a 95% confidence interval) to yield posttest probabilities in the case of a positive or negative result, respectively. For the purpose of the analysis, the likelihood ratio is expressed in logarithmic form. (b) The likelihood matrix on WCE uses deep learning to identify polyps, where studies in the upper left quadrant of the plot can confirm or exclude polyps. Each circle indicates an individual study, and the red diamond represents the summary LRP and LRN for the index test with a 95% confidence region. LRP, likelihood ratio positive; LRN, likelihood ratio negative.

The pooled sensitivity and specificity of WCE for identifying polyps by deep learning were 0.97 (95% CI, 0.95–0.98) and 0.97 (95% CI, 0.94–0.98), respectively, in Figure 2. Significant heterogeneity was found in terms of sensitivity and specificity (I^2^ = 88.96%, I^2^ = 94.15%). The combined PLR, NLR, and DOR were 27.19 (95% CI 15.32–50.42), 0.03 (95% CI 0.02–0.05, Figure 2), and 873.69 (95% CI 387.34–1970.74, Figure 3), respectively, and the I^2^ values for PLR, NLR, and DOR were 90.48%, 88.46%, and 100.00%, respectively, which indicated that there was significant heterogeneity. The WCE identification of polyps had a fairly high accuracy rate, with an AUC value of 0.99 in Figure 3. When the pretest probability is 48%, the probability of polyps in patients with positive results increases to 96%, while the probability of polyps in patients with negative results decreases to 3%. Because the positive likelihood ratio was above 10 and the negative likelihood ratio was below 0.10, WCE uses deep learning to identify polyps with positive results, essentially confirming the diagnosis of polyps, and negative results, essentially excluding them in Figure 4.

### 3.3. Subgroup Analyses and Meta-Regression

There was significant heterogeneity in this meta-analysis, and subgroup analyses and meta-regression were conducted to explore the heterogeneity. Subgroup analysis was performed according to the characteristics of polyps identified by WCE. The subgroup analysis is shown in Table 2. Heterogeneity between studies was high, where the I^2^ index was 88.96% overall for sensitivity. The heterogeneity in sensitivity may be the result of the following factors: dataset size, number of patients, total dataset size, country, method, types of capsule endoscopes, test images, and journal type (*P* < 0.05). Heterogeneity between studies was high, where the I^2^ index was 94.15% overall for specificity. The heterogeneity of specificity may be due to the following factors: number of patients, total dataset size, country, method, and test images (*P* < 0.05). Exploring heterogeneity using the joint model revealed the following factors that may contribute to heterogeneity: dataset size, total dataset size, country, method, and test images (*P* < 0.05).

### 3.4. Publication Bias and Sensitivity Analysis

Deek's funnel plot was used to analyse the potential publication bias of the meta-analysis. Deek's test showed a value of 0.28 (95% CI −110.89–38.22), and this suggested no possibility of publication bias (in Supplementary Information Part II). There was also no significant threshold effect by the Spearman correlation coefficient (Spearman correlation coefficient −0.28; *P*=0.51). In influence analysis, each study had no significant effect on the meta-analysis (in Supplementary Information Part I).

## 4. Discussion

Artificial intelligence, the fourth industrial revolution, is and will continue to have a profound impact on medicine [24]. WCE, a noninvasive endoscopic procedure, will be increasingly used in clinical practice as technology advances. The combination of artificial intelligence and noninvasive WCE will certainly lead to great developments in the diagnosis of digestive diseases. We have, for the first time, systematically evaluated the accuracy of WCE in identifying polyps using deep learning. This meta-analysis demonstrated that WCE had the optimal summary sensitivity of 97% and summary specificity of 97% using deep learning to identify polyps. This study also obtained near perfect results of 0.99 and 873 using the AUC and DOR as composite indicators to evaluate diagnostic accuracy. In addition, WCE using deep learning to identify polyps had a 96% chance of a patient being diagnosed with a polyp if it was positive and only a 3% chance of a patient being diagnosed with a polyp if it was negative.

CRC is a disease with high global morbidity and mortality, and the number of new cases of CRC is expected to increase to 2.5 million worldwide by 2035 [25]. Studies have shown that approximately 90% of all CRC evolve from colorectal polyps, particularly adenomatous and serrated polyps, both of which are precancerous and take an average of 10 years to develop into invasive cancer, a process driven primarily by the accumulation of genetic mutations and epigenetic changes [26]. Early detection and removal of colorectal polyps can effectively prevent the development of CRC and significantly reduce the mortality rate of CRC [27]. In clinical practice, colorectal polyps are usually diagnosed by colonoscopy, WCE, and computed tomography colonography (CTC), with WCE being more readily accepted than colonoscopy (4.2% vs. 1%, *P* < 0.001) [28]. Another method, the CTC, was inferior to the CCE in detecting polyps ≥6 mm and exposed to radiation [29]. In a prospective study, CCE and CTC were performed on 100 patients who were unable to complete colonoscopy, and both tests were performed on the entire colon in 98% of patients. The relative sensitivity of CCE was twice that of CTC for colon polyps larger than 6 mm, and the positive predictive values of CCE and CTC for colon polyps larger than 6 mm were 96% and 85.7%, respectively, which concluded that CCE was better than CTC in terms of diagnostic ability [30].

The size of PillCam Colon 2 Capsule Endoscopy (CCE-2) is 31.5 mm × 11.6 mm, and the working time can exceed 10 hours. After technological innovation, it enters the second generation and has obvious progress in technical parameters. Firstly, there are cameras at both ends of the capsule, and the field of view of each camera is increased from 156° to 172°, with the combined cameras approaching 360°, ensuring a wider view of the colonic mucosa. Secondly, the capsule endoscope image acquisition uses the adaptive frame rate (AFR) mode, which enables intelligent frequency conversion of the image acquisition frequency according to the capsule's movement speed [31]. Flat, nonpolypoid lesions in the colorectum, including laterally spreading tumours, are at high risk of developing high-grade heterogeneous hyperplasia and early cancer and are easily missed on colonoscopy. However, CCE-2 has high sensitivity for the diagnosis of flat lesions. The study showed that the sensitivity of CCE-2 for the diagnosis of 67 colorectal lesions was 84%, 78%, and 88% for >6 mm lesions, flat lesions, and elevated lesions, respectively, with no statistically significant difference between the three groups [32]. CCE-2 is currently the most studied in Europe and is therefore also recommended by the European Society of Gastrointestinal Endoscopy (ESGE) as an option for CRC screening in the general risk population, as well as for those who are unable to complete a colonoscopy, refuse a colonoscopy in high-risk groups, or have a contraindication to a colonoscopy [33]. At this time of a new pneumonia outbreak and a global pandemic, capsule endoscopy has the advantages of single-use instruments without anaesthesia and requiring only one operator, a separate room for the examination, separation of examination and film reading, and the use of the Internet and cloud platform. The risk of cross-infection is significantly lower than that of conventional gastroscopy, making it a safer tool for the detection of gastrointestinal diseases during an epidemic [34]. However, reviewing frames is a time-consuming process as they generate a large number of frames, and there is a risk that important lesions are overlooked.

Viewing these images is a monotonous and time-consuming task that takes approximately 50 minutes to complete [35, 36]. As a comprehensive frontier subject, artificial intelligence is widely used in economics, the military, medicine, and daily life. With the rapid development of AI technology in the medical field, its powerful computing and deep learning capabilities have attracted the attention of people in the medical field [37]. Digestive endoscopy, as an important field of AI image recognition applications, has also received more and more attention. A meta-analysis study showed that WCE used deep learning to diagnose ulcers and bleeding with high diagnostic accuracy, with a sensitivity and specificity of 0.95 and 0.94 for ulcers and 0.98 and 0.99 for bleeding, respectively [38]. The hand-engineered methods algorithm uses a framework algorithm such as a support vector machine or binary classifier to classify the image into a corresponding classification set based on the feature information extracted from the image by using the color texture shape information as the main image extraction feature [9]. Although the hand-engineered methods classification method that recognizes fixed features has a high accuracy rate in detecting various lesions, it always has problems such as insufficient training and testing and imperfect artificial feature design [39–43]. In addition, hand-engineered methods encode only part of the image, ignoring the information inherent in the WCE image [44].

Deep learning has applications in the field of clinical medicine because medical data often contain unstructured information such as images and videos that cannot be easily processed. This unstructured information can be processed by a computer trained to imitate the learning process of the human brain (in Supplementary Information Part V). The deep learning SSAEIM method was used to diagnose polyps with a 98% accuracy rate, which was higher than the following hand-engineered methods and was statistically significant (*P* < 0.01) [16]. WCE uses global features to diagnose polyps with accuracy rates of 65% and 85.9%, while WCE uses local features such as SIFT (scale-invariant feature transform) and LBP (local binary pattern) to characterize image patches to diagnose polyps with an accuracy of 86.7%–89.8% [40, 45, 46]. CNN (convolutional neural network) is the best developed deep learning system, which is in a state of continuous learning like the human brain and can automatically identify and detect target images and analyse them quickly and accurately to improve the diagnosis rate of diseases [47]. In a study containing 255 patients and CNNs trained on 11,300 images, colorectal capsule endoscopy used to detect polyps has demonstrated high sensitivity (97.1%) and specificity (93.3%) [20]. In comparison, our meta-analysis had a similar sensitivity (97%) and higher specificity (97%). Because of its fast detection speed and high detection rate, the system can be applied to large sample size screening, saving time and money for colorectal cancer screening [48]. CNN takes approximately 13 minutes to read a full-length CCE video containing 50,000 frames at a read rate of 66 frames per second [18]. Future deep learning will be devoted to various aspects such as autonomous diagnosis, remote diagnosis, and capsule microscopy quality control and will further improve the advantages of capsule microscopy, such as being noninvasive, painless, and convenient to improve the detection rate of lesions.

This study has several limitations. Firstly, the included studies were retrospective studies, possibly subject to selective bias. Secondly, there was high heterogeneity in the combined diagnostic indicators, which may be due to confounding factors such as different methods of deep learning. Thirdly, some of the included studies had small sample sizes and lacked multicentre studies.

With the rapid development of artificial intelligence technology, deep learning algorithms based on its excellent and powerful computing power in the field of medical imaging continue to improve the accuracy of diagnosis and also gradually free up the doctor's workforce, especially in the face of the huge volume of data capsule endoscopy. However, deep learning for WCE has been mostly at the study stage and has not been used in a large number of clinical applications. The reasons for this are the general lack of training with large data volumes to ensure performance and the lack of prospective clinical studies to further prove reliability. To facilitate the further development of intelligent polyp identification, a strong collaboration between the fields of clinical medicine and engineering is needed to seek the support of evidence-based medical evidence such as more large-scale, multicentre, high-quality, prospective randomized controlled studies.

## Figures and Tables

**Table 1:** Summary of studies in the literature review that used deep learning for WCE to identify polyps.

First author	Year of publication	Country	No. of patients	Dataset size (frames)	Total dataset size (frames)	Annotator's experience	Method	Algorithm	Study design	Center	Speed of frames reading	Types of capsule endoscopes	Types of databases	Test images	Journal type
Yuan	2017	China	35	4000	4000	Expert physicians	Deep learning	SSAEIM	Retrospective	Single	Unclear	PillCam SB WCE	Proprietary	NO	Biocomputational
Sindhu	2017	India	Unclear	401	435	Expert physicians	Deep learning	Neural network	Retrospective	Single	Unclear	PillCam SB WCE	Online	NO	Biocomputational
Yuan	2018	China	62	300	3000	Expert physicians	CNN	RIIS-DenseNet	Retrospective	Single	Unclear	WCE	Proprietary	YES	Biocomputational
Garbay	2019	France	18	3586	11952	Unclear	CNN	SqueezeNet	Retrospective	Single	Unclear	WCE	Proprietary	YES	Biocomputational
Sornapudi	2019	USA	18	82	1800	Expert physicians	CNN	ResNet-101	Retrospective	Single	Unclear	PillCam SB WCE	Proprietary	YES	Biocomputational
Nadimi	2019	Denmark	255	1695	11300	Expert physicians	CNN	ZF-Net	Retrospective	Single	Unclear	PillCam Colon 2	Proprietary	YES	Biocomputational
Blanes-Vidal	2019	Denmark	255	1695	11300	Expert physicians	CNN	AlexNet	Retrospective	Single	Unclear	PillCam Colon 2	Proprietary	YES	Clinical
Saraiva	2021	Portugal	24	728	3387259	Expert physicians	CNN	ImageNet	Retrospective	Single	66 frames/second	PillCam Colon 2	Proprietary	YES	Clinical

WCE, wireless capsule endoscopy.

**Table 2:** Subgroup analysis of diagnostic indices (with a 95% confidence interval).

Subgroup	No. of studies	Sensitivity pooled (95% CI)	*p* value	Specificity pooled (95% CI)	*p* value
Dataset size (frames)					
>1000	4	0.98(0.98–0.99)	≤0.001	0.96 (0.94–0.99)	0.06
<1000	4	0.94(0.92–0.96)		0.97(0.94–1.00)	

No. of patients					
>100	2	0.98(0.96–1.00)	0.01	0.95(0.90–1.00)	0.02
<100	6	0.97(0.95–0.98)		0.97(0.95–0.99)	

Total dataset size (frames)					
>10000	4	0.97(0.96–0.99)	≤0.001	0.94(0.93–0.96)	≤0.001
<10000	4	0.96(0.94–0.99)		0.99(0.98–0.99)	

Country					
Asia	3	0.97(0.94–0.99)	≤0.001	0.99(0.98–0.99)	≤0.001
Europe and America	5	0.97(0.95–0.99)		0.94(0.93–0.96)	

Method					
Deep learning	2	0.97(0.95–1.00)	≤0.001	0.99(0.99–0.99)	≤0.001
CNN	6	0.97(0.95–0.99)		0.94(0.93–0.95)	

Types of capsule endoscopes					
WCE	5	0.97(0.96–0.99)	≤0.001	0.98(0.96–0.99)	0.12
PillCam Colon 2	3	0.96(0.94–0.99)		0.94(0.90–0.98)	

Test images					
No	2	0.97(0.95–1.00)	0.01	0.99(0.99–0.99)	≤0.001
Yes	6	0.97(0.95–0.99)		0.94(0.93–0.95)	

Journal type					
Biocomputational	6	0.98(0.96–0.99)	0.01	0.98(0.96–0.99)	0.32
Clinical	2	0.95(0.91–0.99)		0.93(0.87–0.99)	

WCE, wireless capsule endoscopy; CNN, convolutional neural network.

## Data Availability

The data used to support the findings of this study are included within the article.

## Additional Points

*Originality Statement*. The published studies on the use of deep learning for capsule endoscopy have small sample sizes and different algorithms. The study conducted a meta-analysis of the published studies and found that capsule endoscopy using deep learning has high diagnostic accuracy for identifying polyps, providing new evidence for evidence-based medicine.

## Supplementary Materials

Supplementary Materials Supplementary Information Part I: sensitivity analysis of WCE using deep learning to identify polyps. (a) Goodness-of-fit; (b) bivariate normality; (c) Cook's distance; (d) scatter plot of standardized level-2 residuals. Supplementary Information Part II: publication bias for this meta-analysis. Symmetry test for Deek's funnel plot and each circle represents an independent study. Supplementary Information Part III: summary of the methodological quality of the studies included in this meta-analysis. Red circles indicate high risk of bias, yellow circles indicate uncertain risk of bias, and green circles indicate low risk of bias. Supplementary Information Part IV: the detailed literature search process for this meta-analysis included four databases: PubMed, Embase, the Web of Science, and the Cochrane Library. Supplementary Information Part V: the analogy between deep learning neural networks, simple neural networks, and neuronal signaling pathways is depicted schematically.
