# Supplementary material for: Diagnostic Accuracy of Wireless Capsule Endoscopy in Polyp Recognition Using Deep Learning: A Meta-Analysis
Source: Int J Clin Pract. 2022 Mar 19;2022:9338139. doi: 10.1155/2022/9338139 (PMC9159236; doi:10.1155/2022/9338139)
Supplement: Supplementary Materials — Supplementary Information Part I: sensitivity analysis of WCE using deep learning to identify polyps. (a) Goodness-of-fit; (b) bivariate normality; (c) Cook's distance; (d) scatter plot of standardized level-2 residuals. Supplementary Information Part II: publication bias for this meta-analysis. Symmetry test for Deek's funnel plot and each circle represents an independent study. Supplementary Information Part III: summary of the methodological quality of the studies included in this meta-analysis. Red circles indicate high risk of bias, yellow circles indicate uncertain risk of bias, and green circles indicate low risk of bias. Supplementary Information Part IV: the detailed literature search process for this meta-analysis included four databases: PubMed, Embase, the Web of Science, and the Cochrane Library. Supplementary Information Part V: the analogy between deep learning neural networks, simple neural networks, and neuronal signaling pathways is depicted schematically. [file 9338139.f1.zip › 9338139.f1/Supplementary Information Part III.pdf]

	Risk of Bias				Applicability Concerns		
	Patient Selection	Index Test	Reference Standard	Flow and Timing	Patient Selection	Index Test	Reference Standard
Blanes-Vidal 2019	?	+	+	+	+	+	+
Garbay 2019	?	+	?	+	+	+	+
Nadimi 2019	?	+	+	+	+	+	+
Saraiva 2021	?	+	+	+	+	+	+
Sindhu 2017	?	+	+	+	+	+	+
Sornapudi 2019	?	+	+	+	+	+	+
Yuan 2017	?	+	+	+	+	+	+
Yuan 2018	?	+	+	+	+	+	+

− **High** ? **Unclear** + **Low**
