# Supplementary material for: Diagnostic Accuracy of Wireless Capsule Endoscopy in Polyp Recognition Using Deep Learning: A Meta-Analysis
Source: Int J Clin Pract. 2022 Mar 19;2022:9338139. doi: 10.1155/2022/9338139 (PMC9159236; doi:10.1155/2022/9338139)
Supplement: Supplementary Materials — Supplementary Information Part I: sensitivity analysis of WCE using deep learning to identify polyps. (a) Goodness-of-fit; (b) bivariate normality; (c) Cook's distance; (d) scatter plot of standardized level-2 residuals. Supplementary Information Part II: publication bias for this meta-analysis. Symmetry test for Deek's funnel plot and each circle represents an independent study. Supplementary Information Part III: summary of the methodological quality of the studies included in this meta-analysis. Red circles indicate high risk of bias, yellow circles indicate uncertain risk of bias, and green circles indicate low risk of bias. Supplementary Information Part IV: the detailed literature search process for this meta-analysis included four databases: PubMed, Embase, the Web of Science, and the Cochrane Library. Supplementary Information Part V: the analogy between deep learning neural networks, simple neural networks, and neuronal signaling pathways is depicted schematically. [file 9338139.f1.zip › 9338139.f1/Supplementary Information Part IV (3).docx]

**Search details**

Two authors independently conducted a comprehensive and systematic search of PubMed, Embase, Web of Science, and Cochrane databases, respectively, up to December 8, 2021.

**PubMed**

#1: (convolutional neural network) OR (artificial intelligence) OR (AI) OR (neural networks) OR (computer-aided diagnosis) OR (deep learning) 1,316,397

**Details:**

(("convolute"[All Fields] OR "convoluted"[All Fields] OR "convolutes"[All Fields] OR "convoluting"[All Fields] OR "convolution"[All Fields] OR "convolutional"[All Fields] OR "convolutions"[All Fields] OR "convolutive"[All Fields]) AND ("neural networks, computer"[MeSH Terms] OR ("neural"[All Fields] AND "networks"[All Fields] AND "computer"[All Fields]) OR "computer neural networks"[All Fields] OR ("neural"[All Fields] AND "network"[All Fields]) OR "neural network"[All Fields])) OR ("artificial intelligence"[MeSH Terms] OR ("artificial"[All Fields] AND "intelligence"[All Fields]) OR "artificial intelligence"[All Fields]) OR ("antagonists and inhibitors"[MeSH Subheading] OR ("antagonists"[All Fields] AND "inhibitors"[All Fields]) OR "antagonists and inhibitors"[All Fields] OR "ai"[All Fields]) OR ("neural networks, computer"[MeSH Terms] OR ("neural"[All Fields] AND "networks"[All Fields] AND "computer"[All Fields]) OR "computer neural networks"[All Fields] OR ("neural"[All Fields] AND "networks"[All Fields]) OR "neural networks"[All Fields]) OR ("diagnosis, computer assisted"[MeSH Terms] OR ("diagnosis"[All Fields] AND "computer assisted"[All Fields]) OR "computer-assisted diagnosis"[All Fields] OR ("computer"[All Fields] AND "aided"[All Fields] AND "diagnosis"[All Fields]) OR "computer aided diagnosis"[All Fields]) OR ("deep learning"[MeSH Terms] OR ("deep"[All Fields] AND "learning"[All Fields]) OR "deep learning"[All Fields])

#2: (colon capsule endoscopic images) OR (colon capsule endoscopy) OR (capsule endoscopy) 5,853

**Details:**

(("colon"[MeSH Terms] OR "colon"[All Fields] OR "colonic"[All Fields] OR "colons"[All Fields] OR "colon s"[All Fields] OR "colonal"[All Fields] OR "colonically"[All Fields] OR "colonitis"[All Fields]) AND ("capsule s"[All Fields] OR "capsules"[MeSH Terms] OR "capsules"[All Fields] OR "capsule"[All Fields]) AND ("endoscope s"[All Fields] OR "endoscoped"[All Fields] OR "endoscopes"[MeSH Terms] OR "endoscopes"[All Fields] OR "endoscope"[All Fields] OR "endoscopical"[All Fields] OR "endoscopically"[All Fields] OR "endoscopy"[MeSH Terms] OR "endoscopy"[All Fields] OR "endoscopic"[All Fields]) AND ("image"[All Fields] OR "image s"[All Fields] OR "imaged"[All Fields] OR "imager"[All Fields] OR "imager s"[All Fields] OR "imagers"[All Fields] OR "images"[All Fields] OR "imaging"[All Fields] OR "imaging s"[All Fields] OR "imagings"[All Fields])) OR (("colon"[MeSH Terms] OR "colon"[All Fields] OR "colonic"[All Fields] OR "colons"[All Fields] OR "colon s"[All Fields] OR "colonal"[All Fields] OR "colonically"[All Fields] OR "colonitis"[All Fields]) AND ("capsule endoscopy"[MeSH Terms] OR ("capsule"[All Fields] AND "endoscopy"[All Fields]) OR "capsule endoscopy"[All Fields])) OR ("capsule endoscopy"[MeSH Terms] OR ("capsule"[All Fields] AND "endoscopy"[All Fields]) OR "capsule endoscopy"[All Fields])

#3: (colorectal neoplasia) OR (colon Cancer) OR (colonic Polyps) OR (colorectal polyps) OR (colorectal polyp screening) OR (colorectal Neoplasms) 285,709

**Details:**

("colorectal"[All Fields] AND ("neoplasms"[MeSH Terms] OR "neoplasms"[All Fields] OR "neoplasia"[All Fields] OR "neoplasias"[All Fields])) OR ("colonic neoplasms"[MeSH Terms] OR ("colonic"[All Fields] AND "neoplasms"[All Fields]) OR "colonic neoplasms"[All Fields] OR ("colon"[All Fields] AND "cancer"[All Fields]) OR "colon cancer"[All Fields]) OR ("colonic polyps"[MeSH Terms] OR ("colonic"[All Fields] AND "polyps"[All Fields]) OR "colonic polyps"[All Fields]) OR ("colorectal"[All Fields] AND ("polyp s"[All Fields] OR "polypous"[All Fields] OR "polyps"[MeSH Terms] OR "polyps"[All Fields] OR "polyp"[All Fields])) OR ("colorectal"[All Fields] AND ("polyp s"[All Fields] OR "polypous"[All Fields] OR "polyps"[MeSH Terms] OR "polyps"[All Fields] OR "polyp"[All Fields]) AND ("diagnosis"[MeSH Subheading] OR "diagnosis"[All Fields] OR "screening"[All Fields] OR "mass screening"[MeSH Terms] OR ("mass"[All Fields] AND "screening"[All Fields]) OR "mass screening"[All Fields] OR "early detection of cancer"[MeSH Terms] OR ("early"[All Fields] AND "detection"[All Fields] AND "cancer"[All Fields]) OR "early detection of cancer"[All Fields] OR "screen"[All Fields] OR "screenings"[All Fields] OR "screened"[All Fields] OR "screens"[All Fields])) OR ("colorectal neoplasms"[MeSH Terms] OR ("colorectal"[All Fields] AND "neoplasms"[All Fields]) OR "colorectal neoplasms"[All Fields])

#4: #1 and #2 and #3 39

**Embase**

#1: convolutional AND neural AND network OR (artificial AND intelligence) OR ai OR (neural AND networks) OR ('computer aided' AND diagnosis) OR (deep AND learning) 258,208

#2: colorectal AND neoplasia OR (colon AND cancer) OR (colonic AND polyps) OR (colorectal AND polyps) OR (colorectal AND polyp AND screening) OR (colorectal AND neoplasms) 247,182

#3: colon AND capsule AND endoscopy OR (capsule AND endoscopy) OR (colon AND capsule AND endoscopic AND images) 12,436

#4: #1 and #2 and #3 46

**Web of science**

#1: colon capsule endoscopic images (Topic) or colon capsule endoscopy (Topic) or capsule endoscopy (Topic) 9,674

#2: convolutional neural network (Topic) or artificial intelligence (Topic) or AI (Topic) or neural networks (Topic) or computer-aided diagnosis (Topic) or deep learning (Topic) 982,167
#3: colorectal neoplasia (Topic) or colon cancer (Topic) or colonic Polyps (Topic) or colorectal polyps (Topic) or colorectal polyp screening (Topic) or colorectal neoplasms (Topic) 334,392

#4: #1 and #2 and #3 56

**Cochrane Library**

#1: (convolutional neural network):ti,ab,kw OR (artificial intelligence):ti,ab,kw OR (AI):ti,ab,kw OR (neural networks):ti,ab,kw OR (computer-aided diagnosis):ti,ab,kw 8,996

#2: (deep learning):ti,ab,kw 1,051

#3: #1 or #2 9,537

#4: (colon capsule endoscopic images):ti,ab,kw OR (colon capsule endoscopy):ti,ab,kw OR (capsule endoscopy):ti,ab,kw 808

#5: (colorectal neoplasia):ti,ab,kw OR (colon cancer):ti,ab,kw OR (colonic Polyps):ti,ab,kw OR (colorectal polyps):ti,ab,kw OR (colorectal polyp screening):ti,ab,kw 8,998

#6: (colorectal neoplasms):ti,ab,kw 8,305

#7: #5 or #6 14,869

#8: #3 and #4 and #7 0
